# Deep, Surface, or Both? A Study of Occupational Therapy Students' Learning Concepts

**DOI:** 10.1155/2018/3439815

**Published:** 2018-08-07

**Authors:** Tore Bonsaksen

**Affiliations:** ^1^Department of Occupational Therapy, Prosthetics and Orthotics, Faculty of Health Sciences, OsloMet-Oslo Metropolitan University, Oslo, Norway; ^2^Faculty of Health Studies, VID Specialized University, Sandnes, Norway

## Abstract

**Background:**

Students' conceptualization of learning has been associated with their approaches to studying. However, whether students' learning concepts are associated with their personal characteristics is unknown.

**Aim:**

To investigate whether sociodemographic, education-related, and personal factors were associated with the learning concepts of Norwegian occupational therapy students.

**Methods:**

One hundred and forty-nine students (mean age 23.9 years, 79.2% women) participated in the study. The employed self-report questionnaires included the Approaches and Study Skills Inventory for Students, the Rosenberg Self-Esteem Scale, and the General Self-Efficacy Scale. Differences between student cohorts were analyzed with one-way analyses of variance and *χ*^2^ tests, whereas factors associated with the students' learning concepts were analyzed with bivariate correlation and linear regression models.

**Results:**

The students' mean scores on the deep and surface learning concept scales were similar. Spending more time on the independent study was associated with having higher scores on the unidimensional learning concept measure.

**Conclusions:**

The students' learning concept appears to encompass a surface concept as well as a deep concept of learning, and the two ways of conceptualizing learning were positively related to each other. Over time, a mature deep concept may add to, rather than replace, a basic surface concept of learning.

## 1. Introduction

According to Biggs [1], the factors important for students' learning in higher education can be organized according to the presage, process, and product of learning—the three P model. The presage factors are those that constitute the student's readiness for the tertiary educational experience, emphasizing intellectual inquiry, logical reasoning, and the development of a comprehensive understanding of the phenomena under scrutiny. These factors include the student's background characteristics but also the situational context within which learning takes place [1]. The process factors are concerned with how the student engages with the study materials. A substantial amount of educational research has built on the concepts of deep, strategic, and surface approaches to studying, denoting a typology of different attitudes and behaviors when engaged in studying [2, 3]. Finally, the product or outcome of learning is considered to be a blend of theoretical knowledge, practical skills, and generic competence [4]. For practical purposes, much research has operationalized “learning outcome” as exam grades, although for students of occupational therapy and other healthcare professions, professional skill acquisition should be considered of equal importance [5].

A substantial amount of educational research suggests a relationship between the learning process and the subsequent learning product. Students' approaches to studying have been shown to play a significant part in determining learning outcomes, as frequently measured by their academic performance results. Specifically, researchers have found a relatively consistent pattern of associations between employing deep and strategic study approaches and achieving good academic grades [5–10]. Conversely, using a surface approach to studying has been associated with poorer academic grades.

In view of the detected relationships between productive study approaches and subsequent learning outcomes, researchers have also examined how the presage factors may have an impact on the students' learning process, often conceptualized as their approaches to studying. Among the individual student factors, higher age has been found to be associated with the deep and strategic study approaches [11–13], whereas the impact of gender has been ambiguous [11]. Other personal factors, like general self-efficacy [13] and intrinsic motivation [14], have also been shown to be associated with productive study approaches among undergraduate students of occupational therapy and psychology, respectively. Much research attention has been devoted to exploring relationships between the learning environment and the students' approaches to studying (e.g., [15–17]). An illustrating example was provided by Sun and Richardson [18] in their study of British business candidates. Their results showed that all subscales of a course experience questionnaire (e.g., appropriate assessment and workload, clarity of goals and standards, and good teaching) were positively associated with deep and strategic approaches to studying and negatively associated with a surface approach.

Notwithstanding the demonstrated impact of the learning environment, Richardson [19] argued that a substantial variance proportion related to approaches to studying was still left unexplained, even after adjusting for perceptions of the learning environment. Considering other factors that might contribute to explain students' variations in study approaches, he pointed towards another presage factor—the students' own conceptions of learning. In support of this reasoning, early research demonstrated relationships between “reproductive” conceptions of learning (i.e., the belief that learning is mainly concerned with the ability to remember facts) and a surface approach to studying and conversely, between a “reconstructive” concept of learning (i.e., the belief that learning is concerned with understanding things differently) and a deep approach to studying [20]. Dart and coworkers [21] similarly found that students who conceived learning as understanding, perceiving something in a different way, personal fulfilment, and developing social competence were more inclined to use a deep approach to studying. Conversely, students who conceived learning predominantly as increase in knowledge, remembering, and reproducing were more inclined to use a surface approach to studying. Sharma [22] found that the majority of his sample of accounting students conceived learning to be about acquiring knowledge rather than developing their understanding and expressed concern in the anticipation of what could be called “robotic” accountants.

However, there is reason to suggest that students' conceptions of knowledge are not either deep or surface but that they evolve into more sophisticated forms over time. Building on the ideas of William Perry, Entwistle and Peterson [23] described a typical course of learning concept development. Starting out from dualism, the student is inclined to think that something is right or wrong. The next phase, multiplicity, indicates that the student accepts there exist several views on an issue. Entering the relativism phase indicates that the student has arrived at an understanding of the different views as depending on the person's interpretation of evidence. Commitment within relativism, the final phase in the model, involves making a personal stand, while accepting other views to be concurrently valid. In support of such a view, the recent study of occupational therapy students found that those with broader conceptions of learning also had higher scores on the deep and strategic study approach scales, compared to their counterparts with narrower conceptions of learning [24].

Applying this model to occupational therapy education, a student may for example initially feel certain that group-based activities are good for persons with depression (dualism): depressed persons need to break out from isolation. He or she may then accept that others have different opinions on the matter (multiplicity): group activities can be overwhelming and may stimulate feelings of inferiority in relation to others. Later, the student may realize that the assessment of potential benefits and risks of group activities for depression depend on the employed perspective (relativism) and may choose to become more involved in one such perspective while still acknowledging the perspective of others (commitment within relativism).

To summarize, a long line of education research has taken the students' approaches to learning (SAL) tradition as the point of departure. There is a need for research oriented towards relevant outcomes but also research that explores how the different elements of the educational setting—presage, process, and product—work together [25]. In this study, Richardson's [19] view that students' learning conceptions are of importance for both the process and the outcomes of learning is pursued further. To date, it appears that no such study of learning conceptions has been conducted with occupational therapy students. Given the importance of students' learning concepts, an inquiry into their associated factors would allow for a more comprehensive understanding of their role in occupational therapy students' learning. The research question guiding the current study is the following: What are the relationships between occupational therapy students' sociodemographic, personal, and education-related characteristics and their concepts of learning?

## 2. Methods

### 2.1. Design and Setting of the Study

A cross-sectional design was used. The study was conducted in the context of a cross-cultural study, which included four different countries [26]. In the current study, only the data from the Norwegian students were used.

### 2.2. Participants and Recruitment

For inclusion in the study, the students needed to be enrolled in the university's undergraduate occupational therapy education program and provide informed consent to participate. Students from all three-year cohorts participated in the study. The questionnaires were distributed to the students during breaks in classes in January 2015.

### 2.3. Measurement

#### 2.3.1. Sociodemographic Factors

Data regarding age (years) and gender (male = 0, female = 1) were collected by questionnaire.

#### 2.3.2. Personal Factors

The *Rosenberg Self-Esteem Scale* (RSES; [27]) was used to assess self-esteem. The original RSES has ten items, with responses ranging 1–4 (“strongly agree” to “strongly disagree”). One example item is “I take a positive attitude toward myself.” A Norwegian version with four items (RSES-4) was used in this study, and the scale consisting of the four extracted items was strongly correlated (*r* = 0.95) with the full 10-item version [28, 29]. The RSES-4 sum score ranges 4–16, with higher score representing higher self-esteem. In the Norwegian sample, Cronbach's *α* was 0.67 [30], which is lower than the internal consistency shown in other Norwegian studies [31, 32].

The *General Self-Efficacy Scale* (GSE) [33] measures optimistic self-beliefs about coping with a variety of challenges and demands in life. The scale's 10 items are each rated 1–4 (“not at all true” to “exactly true”), and a sum score is calculated by adding the scores on the ten items. The GSE score range is therefore 10–40, with higher scores indicating higher general self-efficacy. One example item is “I can solve most problems if I invest the necessary effort.” Psychometric studies of the GSE have consistently produced a one-factor solution [34, 35], and internal consistency (Cronbach's *α*) of the GSE scale was 0.86 [30], which is considered very good [36, 37].

#### 2.3.3. Education-Related Factors

Data related to the students' learning concepts were obtained from the Approaches and Study Skills Inventory for Students (ASSIST; [2, 38]). In this study, we used a Norwegian instrument translation [39] that has been psychometrically examined within the same sample [40].

The first part of the ASSIST, concerned with the conceptions of learning, consists of six statements. To each of these statements, the respondent rates his or her level of agreement on a 1–5 scale. A rating of “1” means the statement content is “very different” from the student's own thinking, whereas a rating of “5” means it is “very close” to the student's own thinking. The conceptions of learning were originally considered to be of two different kinds [2, 41]: learning conceived as a process of reproducing information (surface concept) and as a process of constructing personal understanding and meaning (deep concept). However, a recent study of the psychometric properties of the learning concepts reflected in these items concluded that the six items might preferably constitute a unidimensional scale, with all six items reflecting aspects of one higher-order concept of learning [40]. Factor loadings for the one-factor measure, tentatively labeled “collected efforts,” ranged between 0.42 and 0.76, and internal consistency of the scale items was *α* = 0.70. For the deep learning concept, factor loadings ranged between 0.54 and 0.81 and internal consistency was *α* = 0.61. For the surface learning concept, factor loadings ranged between 0.67 and 0.78 and internal consistency was *α* = 0.61. The collected efforts' measures, in addition to the deep and surface concept measures, were used as dependent variables in the current study.

One last item of the ASSIST [2, 38] asks the students to think of the grades they have obtained and then perform an overall self-assessment in terms of how well they have been doing in the course so far. Students rated themselves on a 1–9 scale, where 1 indicated “rather badly”, 3 “not so well”, 5 “about average”, 7 “quite well”, and 9 “very well.”

Each participant was registered as belonging to one of the three-year cohorts. Previous higher education experience was dichotomized into two categories: having prior education from university or college (coded 1) versus not having any prior education from university or college (coded 0). The average number of weekly hours spent on the independent study was registered as a continuous variable.

### 2.4. Data Analysis

All data were entered into the computer program IBM SPSS version 24 [42]. Descriptive analyses were performed on all variables using means (*M*), standard deviations (SD), frequencies, and percentages as appropriate. Differences between students in different study cohorts were examined with chi-squared tests (for categorical variables) and with one-way analyses of variance (for continuous variables). When conducting multiple comparisons between student cohorts, the Tukey honest significant difference (HSD) correction was applied to adjust for inflating error rates.

Bivariate associations between variables were assessed with Pearson's correlation coefficient *r*. To assess the extent to which the three learning concept measures could be explained by the independent variables, three subsequent hierarchical linear regression analyses were performed. These analyses also assessed the strength of the independent associations between each of the independent variables and the participants' learning concept. The hierarchy of each of the regression models adhered to Bonsaksen's previous modeling [43]: (1) age and gender, (2) self-esteem and general self-efficacy, and (3) cohort, prior higher education, average time per week spent on the independent study, and self-assessment of study performance. Effect sizes (ES, *r*, and *β*) were interpreted according to Cohen [44]: small ES = 0.10, medium ES = 0.30, and large ES = 0.50. The level of statistical significance was set at *p* < 0.05.

### 2.5. Ethics

Approval for conducting the study was obtained from the Norwegian Centre for Research Data (project number 40314). The students were informed that completion of the questionnaires was voluntary, that their responses would be kept confidential, and that there would be no negative consequences from opting not to participate in the study.

## 3. Results

### 3.1. Participants

One hundred and sixty students opted to participate in the study. Of these, 149 students had valid scores on all variables used in the current study, and these students constitute the sample. The study sample is described in Table 1. All three-year levels were included (first year students *n* = 51, second year *n* = 49, and third year *n* = 49). The students' mean age was 23.9 years (SD = 4.4 years), and there was a majority of female students (*n* = 118, 79.2%). The sample as a whole had similar scores on the deep learning concept (*M* = 12.8, SD = 1.5), compared to their scores on the surface learning concept (*M* = 12.8, SD = 1.4). There were statistically significant differences between study cohorts regarding age, average time spent on the independent study, and self-assessment of study performance.

### 3.2. Unadjusted Associations with the Learning Concepts

The unadjusted associations between the study variables are shown in Table 2. Being of higher age, having higher general self-efficacy, spending more time on the independent study, and having higher self-assessment of own study performance were associated with higher scores on the deep learning concept. Spending more time on the independent study was also associated with higher scores on the surface learning concept and with higher scores on the combined, unidimensional measure (“collected efforts”). The positive association between the deep and the surface learning concepts was statistically significant and of medium size.

### 3.3. Adjusted Associations with the Learning Concepts

The adjusted associations between the independent study variables and the three learning concepts are shown in Table 3. The deep concept regression model was statistically significant, accounting for 13.7% of the data variance. However, none of the independent variables was significantly associated with scores on the deep concept, while controlling for all variables. The surface concept regression model was not statistically significant, accounted for 6.0% of the variance, and no direct associations were detected. The collected efforts' regression model accounted for 10.0% of the variance. It was not statistically significant, but spending more time on the independent study was directly associated with higher scores on the concept.

## 4. Discussion

The aim of the current study was to examine associations between sociodemographic factors, personal factors, education-related factors, and learning concepts among Norwegian occupational therapy students. Four variables—higher age, higher general self-efficacy, more time spent on the independent study, and higher self-assessment of study performance—showed bivariate associations with the deep concept. Time spent on the independent study showed bivariate associations with all three learning concepts. However, most of the associations vanished when controlling for all variables in the multivariate analyses.

The bivariate analyses of factors associated with the deep learning concept showed the same pattern as shown in previous studies, in which the deep approach to studying [24] and a measure of preferences for teaching “supporting understanding” [43] were used as outcomes (Table 2). The shared pattern of associations lend support to the notion that these aspects of learning are interrelated. Entwistle and Peterson [23], for example, suggested the occurrence of two-way relationships between learning conceptions and study approaches. Conceptions might be developed from experiences of teaching and studying and may then influence subsequent ways of studying. An unpublished factor analysis, including learning concept items and study approach items together, similarly linked a deep concept of learning with study behaviors that reflect a deep approach to studying [45]. Thus, the unadjusted analysis suggest that some of the same factors that contribute to shape a deep approach to studying [13, 24] and a matching preference for courses and teaching [43] may also contribute to explain variations in the deep concept of learning.

However, the associations with the deep concept scores vanished when statistically controlling for all variables in the multivariate regression analysis. This is in contrast to the study of factors associated with teaching preferences [43]. In that study, age, general self-efficacy, and time spent on the independent study were significantly associated with the teaching preference “supporting understanding,” while controlling for the exact same set of variables as in the current study. The different results may indicate that while some factors stand out as individually associated with a deep-related teaching preference, a more complex pattern of interrelationships between several factors may cancel each other out when attempting to relate the scores to a deep learning concept. Further studies may investigate other variables of interest, as well as combinations of variables, that may contribute to explain variations in deep learning concept scores.

More time spent on the independent study remained associated with higher scores on the unidimensional learning concept measure, “collected efforts,” after adjusting for the effect of all variables. The meaning of this finding is that more persistent study behaviors occurred among students who had a broader view of learning, that is, among those who agreed more strongly to more of the items on the learning concept measure. Students with a narrower view of learning spent less time studying. Previous research has shown that time spent on independent studying is important for subsequent academic performance among occupational therapy students, regardless of their study approaches [5]. However, the recent study also showed that a broader learning concept was significantly associated with higher scores on both deep and strategic approaches to studying [24]. Taken together, these variables may be related in a cyclical pattern. More time spent on the independent study appears to be related to a broader view of what learning is (Table 3) and also to using productive approaches to studying [13]. Conversely, a broader view of learning has been shown to be associated with productive study approaches [24] and may also make spending time on studies more attractive to students. However, the cross-sectional design of the current study precludes us from being conclusive about the direction of these relationships. Further studies should preferably investigate learning concepts, preferences for teaching, and approaches to studying prospectively and in a longitudinal perspective. That way, researchers will be better positioned to clarify the nature of associations and to predict outcomes at subsequent time points.

Considering the scale range (3–15) for both of the deep and the surface learning concepts, the students had high and similar scores on both concept measures (Table 1). Moreover, the two scales for measuring learning concepts were positively correlated (Table 2). The positive association was unexpected, as theory tends to emphasize their differences and not their possible similarities [2, 23, 41]. On the other hand, we notice that Buckley and coworkers [46] found an even stronger positive association (*r* = 0.60, *p* < 0.01) between the deep and surface concept measures used in their study. Further, a recent factor analysis found that a one-factor structure, comprised by all learning concept items together, was a better fit to the data than the two-factor solution [40].

According to the above, the deep and surface learning concepts may not be as different from one another as theory might suggest. In view of William Perry's work, as described by Entwistle and Peterson [23], we may rather think of them both as constituents of a learning concept that develops over time. In the first two developmental phases (dualism and multiplicity), the student may be strongly rooted in a surface concept of learning. This is the phase where he or she wants to get the facts straight and be sure to remember the right answer. In the next two phases (relativism and commitment within relativism), the student needs to incorporate a view that knowledge depends on interpretations. Taking into consideration the results of this study, this development may not imply abandoning the surface concept of learning. However, incorporating a view of learning as something that extends the mere acquisition of facts is required in higher education. Thus, we may assume a line of development starting with a surface-based learning concept, which then gradually incorporates a “deeper” concept according to a growing awareness of the difference between empirical facts and theoretical understanding. In line with critique relating to an overly simplistic dichotomy between deep and surface approaches to studying [47], we may apply a similar critique on the issue of students' ways of conceptualizing learning. Such differences appear to be a matter of nuance rather than a clear-cut dichotomy.

Last, a few comments go beyond the immediate scope of the current study. The study is situated in a context where more research related to occupational therapy education is explicitly called for. The call is not just related to establishing evidence for instructional methods but also to learn more broadly about who occupational therapy students are and how and why they learn [25, 48, 49]. The current study goes beyond these questions in its investigation of the even more fundamental question: the students' idea of what learning is all about. Following Hooper's [50] idea of merging AOTA's research agenda [51] with their education research agenda [52], the study could be classified as basic research concerned with learner characteristics and competencies. Although basic research is based on a long-standing tradition, as it is concerned with describing, clarifying, and testing concepts relevant for the profession, basic research concerned with occupational therapy students seems to be less well developed. Consequently, this study adds to the state of knowledge related to the learning concepts among the students in our own profession.

## 5. Study Limitations

The study is limited by a relatively small sample. The participants were recruited by convenience, and they all came from one particular education program at one particular university in Norway. Thus, generalizations should be performed with much caution. Further, the cross-sectional design of the study prohibits inferring cause and effect relationships; thus, the directions of the detected associations are uncertain and may also be cyclical in their nature. A number of statistical tests were performed, and with an increasing number of tests, the probability of obtaining false results also increase (type I error; [53]).

All data were collected using self-report questionnaires, which is frequently used in this field of research. However, one should also be aware that study approach questionnaires provide data that may or may not fit well with the students' own narrative. For example, one mixed-method study found that the study process questionnaire data from teaching students in Spain suggested that they had a largely deep, nonsurface approach to learning [54]. However, judging from the students' own narratives (data derived from the responses to one open-ended question about how they learned), their study approach was instead surface-based. Such inconsistencies between findings derived from quantitative and qualitative data also suggest caution when interpreting the results of this and similar studies.

## 6. Conclusion and Implications

This study found similar levels of deep and surface conceptions of learning among the Norwegian occupational therapy students. The two theoretically derived concepts were positively associated, suggesting that some of their characteristics are shared rather than different from one another. A clear-cut dichotomy between the deep and the surface learning concepts appears not to be appropriate, rather a gradual development, where the deep concept over time adds to and expands the more basic surface concept, is suggested. Students who spent more time on independent studies had a more broadly composed learning concept, compared to their counterparts. In the future, studies of occupational therapy students' learning concepts should be conducted in a longitudinal perspective.

For occupational therapy educators, the study has several implications. First, understanding the background for students' learning concepts is not a straightforward task. Several sociodemographic, personal, and education-related variables were included as explanatory factors in this study, but only a small proportion of the outcome variance was accounted for. Thus, educators should not worry if they feel they do not fully understand what makes their students think about learning the way they do—a state of mild confusion in this area may seem to reflect the state of the art. Second, students may sustain the idea that learning is about acquiring facts and pieces of information, and obviously, being able to remember facts is not a problem in itself. Problems arise if and when students believe that learning is synonymous to memorizing and comprises nothing else. Thus, educators may try to develop a patience toward students they believe is too hung up on memorizing, and rather aim to assist them in widening their perspective of what learning is—not necessarily try to make them abandon their original ideas. This study suggests that the wider perspective of learning builds on the narrower view but does not replace it. Lastly, a broader view of learning appears to be related to spending more time on the independent study. Educators may therefore guide and motivate students to engage in independent studies. In turn, engaging in independent learning activities may add to the students' ideas of what learning is altogether.

## Figures and Tables

**Table 1:** Characteristics of the study sample (*n* = 149).

Variables	Total sample (*n* = 149)	1st year (*n* = 51)	2nd year (*n* = 49)	3rd year (*n* = 49)	*p*
Sociodemographic factors					
Age, *M* (SD)	23.9 (4.4)	22.9 (4.4)	23.0 (2.4)	25.7 (5.2)	0.001
Female gender, *n* (%)	118 (79.2)	42 (82.4)	36 (73.5)	40 (81.6)	0.48
Personal factors					
Self-esteem, *M* (SD)	12.3 (1.9)	12.1 (1.9)	12.5 (1.8)	12.5 (1.9)	0.38
General self-efficacy, *M* (SD)	28.4 (5.0)	27.6 (5.0)	28.8 (4.9)	28.9 (5.3)	0.37
Education factors					
Prior higher education, *n* (%)	65 (43.6)	22 (43.1)	22 (44.9)	21 (42.9)	0.98
Average time per week on the independent study, *M* (SD)	9.3 (5.0)	11.0 (4.1)	6.7 (3.6)	10.2 (6.7)	<0.001
Self-assessment of study performance, *M* (SD)	6.2 (1.3)	5.7 (1.5)	6.3 (1.2)	6.5 (1.2)	<0.01
Learning concepts					
Deep concept, *M* (SD)	12.8 (1.5)	12.7 (1.5)	12.9 (1.3)	12.9 (1.6)	0.85
Surface concept, *M* (SD)	12.8 (1.4)	13.1 (1.5)	12.4 (1.5)	12.9 (1.3)	0.08
Collected efforts' concept, *M* (SD)	25.6 (2.3)	25.8 (2.4)	25.3 (2.3)	25.6 (2.3)	0.50

*Note*. Statistical tests are chi-squared test and one-way ANOVA.

**Table 2:** Bivariate associations between the study variables (*n* = 149).

Variables	Deep concept		Surface concept		Collected effort concept	
	*r*	*p*	*r*	*p*	*r*	*p*
Age	0.18	<0.05	−0.03	0.76	0.10	0.25
Gender	0.14	0.10	0.12	0.16	0.15	0.06
Self-esteem	0.12	0.14	−0.00	0.98	0.08	0.36
General self-efficacy	0.19	<0.05	−0.06	0.45	0.08	0.32
Cohort	0.04	0.60	−0.07	0.39	−0.02	0.84
Prior higher education	−0.05	0.54	0.09	0.27	0.02	0.77
Average time per week on the independent study	0.24	<0.01	0.17	<0.05	0.25	<0.01
Self-assessment of study performance	0.17	<0.05	0.05	0.54	0.14	0.10
Surface concept	0.29	<0.001				

*Note*. Statistical test is Pearson correlation coefficient *r*. Coding of categorical variables: male = 0; female = 1. No prior higher education = 0; prior higher education = 1. For all other variables, higher scores indicate higher levels.

**Table 3:** Hierarchical linear regression analyses showing direct associations with the students' learning concepts (*n* = 149).

Independent variables	Deep concept		Surface concept		Collected effort concept	
	*β*	*p*	*β*	*p*	*β*	*p*
Sociodemographic factors						
Age	0.14	0.11	−0.08	0.41	0.04	0.66
Gender	0.17	0.06	0.08	0.36	0.16	0.08
*Explained variance*	**4.5%**	**0.03**	**1.4%**	**0.35**	**3.1%**	**0.10**
Personal factors						
Self-esteem	0.05	0.61	0.07	0.53	0.08	0.48
General self-efficacy	0.15	0.15	−0.11	0.32	0.03	0.80
*R ^2^ change*	**5.2%**	**0.02**	**0.6%**	**0.65**	**2.1%**	**0.21**
*Explained variance*	**9.7%**	**<0.01**	**2.0%**	**0.56**	**5.1**	**0.11**
Education factors						
Cohort	−0.03	0.73	−0.06	0.54	−0.05	0.55
Prior higher education	−0.10	0.24	0.09	0.31	−0.01	0.93
Average time per week on the independent study	0.15	0.09	0.16	0.08	0.19	<0.05
Self-assessment of study performance	0.11	0.20	0.08	0.39	0.12	0.18
*R ^2^ change*	**4.0%**	**0.17**	**3.9%**	**0.22**	**4.8%**	**0.12**
*Explained variance*	**13.7%**	**<0.01**	**6.0%**	**0.36**	**10.0%**	**0.06**

*Note*. Table content is standardized beta weights and corresponding *p* values. Coding of categorical variables: male = 0; female = 1. No prior higher education = 0; prior higher education = 1. For all other variables, higher scores indicate higher levels.

## Data Availability

The data used to support the findings of this study are available from the corresponding author upon reasonable request.
